# Expanding the chemical exposome using in-silico transformation analysis – an example using insecticides

**DOI:** 10.1101/2025.09.04.674275

**Published:** 2025-09-09

**Authors:** Mannivannan Jothiramajayam, Dinesh Barupal

**Affiliations:** 1 Integrated Data Science Laboratory for Metabolomics and Exposomics, Department of Environmental Medicine, Icahn School of Medicine at Mount Sinai, New York, USA

**Keywords:** Metabolomics, Exposome, Computational Chemistry, In-silico metabolism, ISTA, RDChiral, RXNMapper, Rxn-INSIGHT, reaction databases, Insecticides

## Abstract

The exposome encompasses a vast chemical space that can originate from the consumer industry and environmental sources. Once these chemicals enter into cells (human or other organisms), they can be also transformed into products that differ in terms of toxicity and health effects. Recent developments in machine learning methods and chemical data science resources have enabled the in-silico prediction of transformation reactions. Here, we report an integrated workflow of these existing resources (RXNMmapper, Rxn-INSIGHT and RDChiral) to predict the transformation product for any chemical in the exposome. We have generated a large library of reaction templates from > 90,000 reactions sourced from the PubChem database. Utility of the reaction screening and transformation prediction workflow has been demonstrated for insecticides structures (n=181), yielding 21,284 unique transformation products. Many of these products have PubChem entries but have not yet been linked with the parent compounds. By identifying a multitude of plausible new products, this approach may expand our understanding of the metabolism of chemical exposure, guide future biomonitoring research and characterize of the human exposome in the existing datasets and chemical databases.

## Graphical Abstract

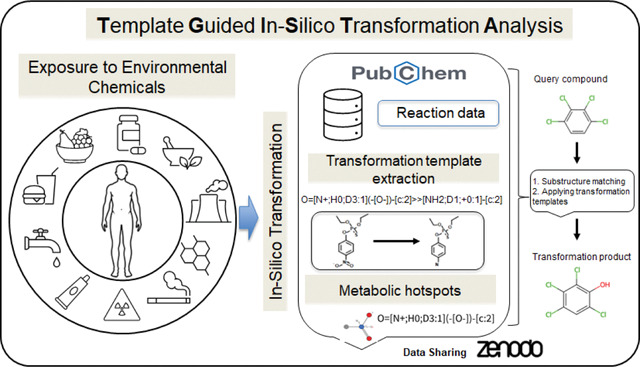

## Introduction:

The chemical exposome represents the totality of an individual’s chemical exposures from all sources throughout their lifespan^1, 2^. This concept provides a comprehensive framework for understanding how the environment influences human health and diseases^3–7^. Human biomonitoring programs using targeted and untargeted assays have been instrumental in quantifying and cataloguing the body burden of chemical exposome^8–10^. These efforts have demonstrated widespread exposure to various classes of chemicals, including pesticides, food-additives, industrial contaminants, and environmental toxins. Once these chemicals enter the human body, they may get transformed by xenobiotic metabolism and other enzymatic reactions, generating additional toxic chemicals^11–14^. Targeted and untargeted biomonitoring assays rely on existing chemical exposome databases such as the Blood Exposome Database^15^ for finding high priority chemicals that are expected in a human biospecimen. Although, the database catalogues over 50,000 chemicals, the vast space of the transformation products, which is the “dark matter” of the chemical exposome, remains largely unexplored.

To expand the chemical exposome, we can use computational methods^16^ to predict the transformation products for an exposure chemical. Such in-silico approach is often used in drug discovery pipelines to identify potential drug metabolites for preclinical toxicity studies^17, 18^. Similarly, in-silico transformation analysis (ISTA) for exposome chemicals can contribute to discoveries of new xenobiotic metabolic pathways and generate new hypotheses regarding how chemical exposome is processed inside the body and how it can affect cellular pathways.

A range of existing computational resources can be integrated into a framework for ISTA, including PubChem^19^ for chemical information including reaction data, RXNMapper^20^ for computing atomic mapping of reaction and RDChiral^21^ for extracting reaction templates. A reaction template captures the transformation patterns within an atom-mapped reaction which can be applied to a chemical structure to generate a new product. We can generate hundreds of new in-silico chemicals by utilizing the large-volume of reaction data from PubChem. Many of these chemicals might be already known structures but not yet linked with a known reaction.

**Figure 1.**
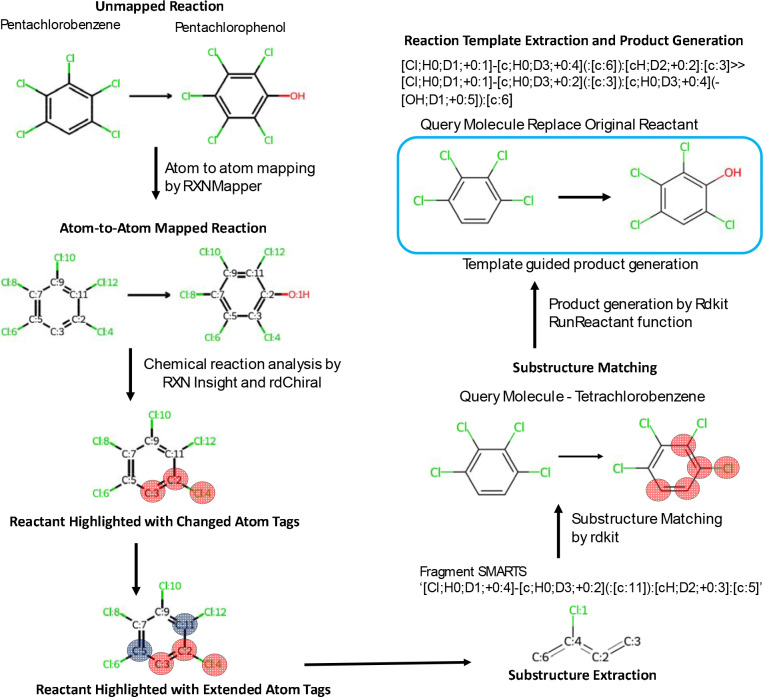
Template guided In-silico transformation analysis (ISTA) workflow. The workflow begins with atom-to-atom mapping of archived chemical/biochemical reactions using RXNMapper. From these mapped reactions, reactive sites, atoms that undergo changes during the reaction were identified, and corresponding substructures were extracted from the reactants. The chemical transformation templates that define transformation patterns were generated from atom-to-atom mapped reaction using the rdChiral, a python library. For a given query compound, substructure matching was performed using RDKit and corresponding transformation template was applied to generate potential transformation products. The workflow is exemplified by the transformation of pentachlorobenzene into pentachlorophenol.

In this study, we have utilized the PubChem reaction data to create a large-database of reaction templates and then we have established an in-silico transformation analysis workflow (Figure 1) using existing methods and applied it to known insecticide structures. By integrating in-silico prediction with database validation and computational assessment of ADME-Tox (Absorption, Distribution, Metabolism, Excretion, and Toxicity) properties, including bioavailability, metabolic stability, clearance rates, and potential for bioaccumulation, this study presents a useful framework for advancing our understanding of toxicokinetics and systemic exposure profiles. We have demonstrated the potential of this workflow in generating highly plausible transformation products for exposome chemicals, using insecticides as a case study.

## Materials and Methods:

### Reaction Data Preparation:

Reaction data were sourced from the National Center for Biotechnology Information (NCBI) PubChem database (https://pubchem.ncbi.nlm.nih.gov/), a publicly available repository of chemical information, including structures, properties, and biological activities. Reactions were submitted by different sources including RHEA^22^, Reactome^23^, PharmaKGB^24^ and BioCyc^25^ along with biological organism annotations (Table S1 and S2). The canonical SMILES of all reactants and products in a reaction were concatenated with ‘.’ and then joined using the ‘>>‘ separator to create a reaction SMILES (e.g. “SMILES 1>>SMILES 2”). Redundant reactions were excluded if the same PubChem Compound Identifiers (CIDs) were observed in two or more reactions. Additionally, any reaction with missing values in either reactant CIDs or product CIDs was also excluded from further processing. Reaction data are available at Zenodo accession ( https://zenodo.org/records/15881389, #Table 1)

### Chemical Reaction Analysis:

To analyze chemical reactions particularly reaction classification, naming, capturing transformation patterns, we utilized the Rxn-INSIGHT^26^, a python library which uses Rection SMILES as input. Our goal was to obtain the atom-to-atom mapping (AAM) of reactions computed by the RXNMapper^20^, python library within the Rxn-INSIGHT workflow. Atomic mapping enables tracking of atoms from substrates to products within a reaction, allowing an accurate analysis of chemical transformation data. RXNMapper has been shown to outperform other AAM tools^27^. In an atomically mapped reaction, atom map numbers are assigned in a sequential manner in the context of reaction (i.e. based on how reaction data is constructed), from 1 to the maximum atoms in a reaction (Figure 1). To sanitize a reaction, Rxn-INSIGHT checks valency, aromaticity, kekulization, atom hybridization and formal charges for the individual SMILES within a reaction. The sanitized mapped reaction is considered for further analysis.

To analyze transformation patterns within a reaction, Rxn-INSIGHT library generates a bond-electron (BE) matrix for each molecule of reactants and products in a sanitized mapped reaction. These individual matrices combined to construct an overall BE matrix for reactants and products. Diagonal entries of a chemical’s BE matrix represent the count of free valence electrons (non-bonded) of a heavy atom excluding hydrogen. Off-diagonal entries BE matrix represents bond order between two atoms (i) and (j) with a bond order as follows: 0-no bond, 1-single bond, 1.5-aromatic bond, 2-double bond and 3-triple bond^26^. A reaction center is identified by calculating R-matrix, difference between BE matrices of products and reactants. Next, this R matrix was converted to a transformation matrix (T-matrix) by removing rows and columns with zero values, to identify which heavy atoms are part of reaction centers. A single reaction can have multiple reaction centers. Rxn-INSIGHT uses the T-matrix to classify the reactions based on a predefined set of ten reaction classes including acylation, aromatic heterocycle formation, C–C coupling, heteroatom alkylation and arylation, functional group addition, functional group interconversion, protection, deprotection, oxidation, and reduction and miscellaneous. Rxn-INSIGHT also detects the functional groups of individual reactants and products by matching molecular substructure of predefined list of most common functional groups (n=107) in SMARTS notation. In addition, Rxn-INSIGHT calculated several other characteristics for a reaction, including direct participation of ring reactants in transformation, scaffold, involvement of NOS (nitrogen, oxygen, and sulfur) in reaction center, number of reactants and products, and the identification of unaltered molecules such as reagents, solvents, catalysts that do not undergo structural changes during reaction. Each reaction was uniquely identified by an RXN_ID field and included its reaction representation in unmapped reaction (REACTION_SMILES) and atom-to-atom mapped reactions (MAPPED_REACTION, SANITIZED_MAPPED_REACTION). The output of Rxn-INSIGHT workflow is available in the Zenodo (https://zenodo.org/records/15881389, #Table 2).

### Reaction template and substructure database generation:

To capture changes in the connectivity between products and their corresponding reactants, a reaction template was generated from each sanitized mapped reaction using the RDChiral library^21^. These templates indicate a chemical transformation pattern that describes how reactants converted to products during a chemical reaction. These templates were encoded in the SMARTS format, which captured key reactive substructures and facilitated generalization, allowing for application of these rules to a wide variety of compounds with similar or identical substructures. We extracted substructures representing chemical transformation sites within individual reactants for each sanitized mapped reaction using the RDChiral python library^21^. These substructures were needed to screen the reactions for a new substrate. Next, “get_changed_atoms”, a helper function from the RDChiral library was used to identify changed atoms by comparing local atomic properties such as atomic number, number of hydrogens, formal charge, degree, number of radical electrons, aromaticity, or the identity of neighboring atom- differ between reactants and products. To accurately capture the structural context of a compound’s reactivity, all atoms initially identified as changed atoms were expanded (radius=1) by including their directly connected neighboring atoms. Furthermore, if any of these atoms, whether originally changed or their direct neighbors were part of a predefined list of 30 known functional groups (e.g., alkenes, carbonyls, carboxylic acids), the entire functional group was included in the set of changed atoms. Functional groups were considered part of the reaction center if at least one of their atoms was involved in the set of transformed atoms. This expansion ensures that the chemical context influencing reactivity is preserved, as reactivity often arises from interactions among atoms within their immediate chemical environment. For every reaction, the substructure corresponding to individual reactants was generated as a SMARTS pattern. The substructure SMARTS was annotated with its corresponding SMILES representation, reaction ID, the originally identified changed atoms, and the list of expanded atoms derived from structural context analysis. The substructure and template databases are available at Zenodo (https://zenodo.org/records/15881389, #Table 3).

### In-silico transformation analysis for insecticides:

A total of 181 insecticides (https://zenodo.org/records/15881389, #Table 4) were selected for template-based biotransformation product prediction analysis. These compounds were chosen based on three primary criteria: chemical diversity, agricultural significance, and structural availability. Based on the structural properties, mode of entry, mode of action, and toxicity profiles, the selected insecticides were categorized into twelve different classes (Figure S1) as follows: arsenical insecticides (n=2), carbamate insecticides (n=24), neonicotinoid insecticides (n=7), nereistoxin insecticides (n=3), organochlorine insecticides (n=6), organophosphorus insecticides (n=56), phosphoramido insecticides (n=4), pyrazole insecticides (n=5), pyrethroid insecticides (n=27), acaricides (n=24), nematicides(n=4) and others (n=19). Agricultural significance was assessed by consistent application of insecticides in current farming practices or under regulatory scrutiny due to environmental persistence or toxicity. Additionally, compounds with defined chemical structures and available canonical SMILES were considered for this study. SMILES notation representing chemical structures for selected insecticides was downloaded from PubChem database and validated using the RDKit library to ensure structural consistency and format compatibility.

To predict the transformation of a query molecule, we first perform a substructure match of query molecules against a substructure library containing chemical transformation sites. This screening process identified the reactions and reaction templates relevant to the specific substructure. Then each template was applied to the queried insecticide using the ‘RunReactants’ function in RDkit. The product structures were converted to SMILES strings and queried against the PubChem database to see if they were already known structures. ADME properties for the product structures were computed using the ADMET-AI library^28^.

## Results:

### Chemical Reaction Database and Analysis:

**Figure 2.**
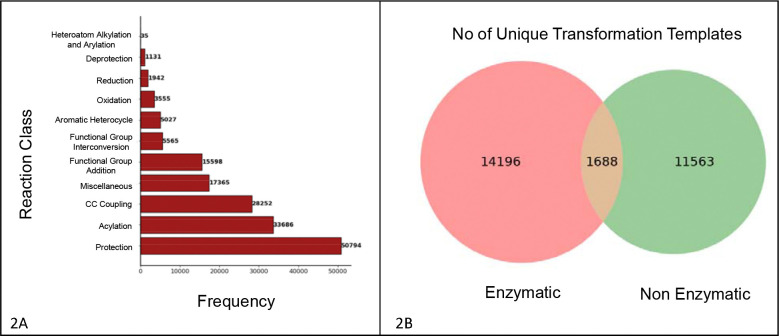
Overview of the reaction data sourced from the PubChem Database. Figure 2A shows the total number of reactions for each reaction class, for which transformation template has been generated. Figure 2B represents distribution of unique chemical transformation templates extracted from enzymatic and non-enzymatic reactions, including the number of overlapping shared between the two categories.

A dataset of 94,658 biochemical reactions was retrieved from PubChem (https://zenodo.org/records/15881389, #Table 1) in April 2025. The Rxn-INSIGHT library was used to categorize each reaction into one of ten major classes. The most prevalent class was protection (n=50,794), followed by acylation (n=33,686), C-C coupling (n=28,252), miscellaneous (n=17,365), and others (Figure 2A). A total of 88,511 reactions had nitrogen, oxygen, or sulfur in their reaction centers. Functional group analysis of both reactants and products revealed the presence of 90 functional groups in reactants and products, with secondary alcohols, ethers, and esters being the most observed in both (https://zenodo.org/records/15881389, #Table 2). 18.4% of reactants and 21% of products contain ring structures; 8.3% of reactions involve changes in ring structure; and 86.4% of molecules were detected with a Markov scaffold. A substantial portion of the reactions were enzymatic (75%), with the remainder being non-enzymatic (Figure 2B). Using the RDChiral library, we extracted the templates for each atom-mapped reaction. The strength of this template-based approach is that the predicted transformations are mechanistically interpretable and supported by known biochemical reactions. To facilitate the faster matching of a query compound, sub-structures that correspond to the metabolic hotspots or reaction centers were extracted for each reaction (See method).

### In-silico transformation analysis for insecticides:

For the present study, 181 insecticides with a wide range of molecular characteristics reflecting significant physicochemical and structural diversity were chosen for template-guided in-silico transformation analysis (https://zenodo.org/records/15881389, #Table 4). These cover historically and currently significant insecticides like parathion, pentachlorophenol, carbaryl, rotenone, and dicofol. Organophosphorus, carbamate, and pyrethroid insecticides formed distinct groups in a tSNE-based chemical diversity visualization using molecular fingerprints. However, there is some overlap between acaricides, others, carbamate, and phosphoramido insecticides, indicating certain overlapping structural characteristics. We expected to identify different chemical reactions that can transform these insecticides due to the chemical diversity.

**Figure 3:**
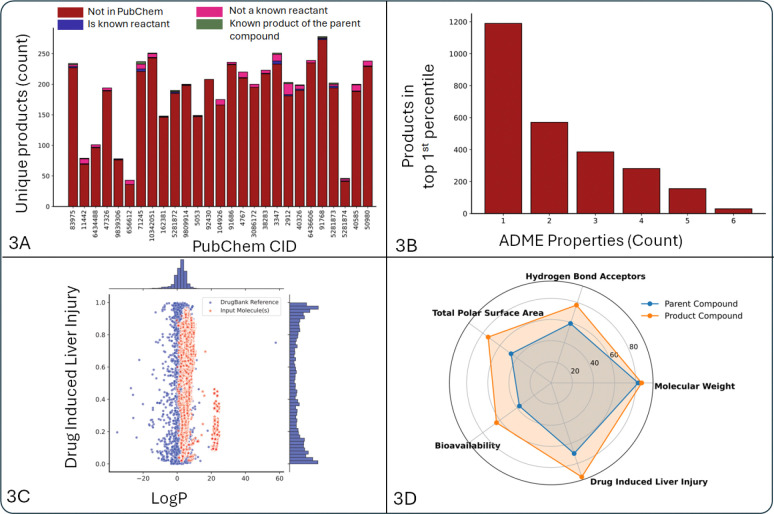
In-silico transformation analysis results. 3A) the number of unique transformation products generated for each query compound in the pyrethroid insecticide class. It indicates whether these products are already known, linked to any reaction in the PubChem database, and the total count of overlapping reactions between the query compound (CID) and its transformation products. 3B) the frequency of transformation products that fall within the top 1 percentile of ADME-Tox properties, compared to reference compounds from DrugBank. 3C) the potential of transformation products to induce drug-induced liver injury (DILI), in comparison with known drugs from DrugBank that have experimentally validated properties. 3D) comparison of various ADME-Tox properties between the top DILI-associated transformation product and its corresponding parent compound.

The established workflow (Figure 1) was applied to a structurally diverse set of 181 insecticides (https://zenodo.org/records/15881389, Table 5) to predict their transformation products. The workflow successfully generated transformation products for 171 of the 181 query compounds. This analysis yielded a total of 21,284 unique transformation products. The number of products generated for each insecticide varied significantly, with a median of 86 unique products per compound (Figure S2). Pyrethroid insecticides were particularly reactive in the model, contributing over 5,000 unique products as a class. Validation against the PubChem database revealed that 1,304 (1.56%) of the predicted products were already known chemical structures, though many had not been previously linked to their parent insecticides. For example, the study identified Nicotine (PubChem CID 89594) as a notable compound, with 62 of its predicted metabolites corresponding to known compounds in PubChem. The ability of the workflow to identify numerous known products for a well-studied compound like nicotine lends confidence to its predictions for less-studied insecticides. Overall, 311 unique PubChem CIDs are associated with at least one reaction across the dataset. Within this subset, a substantial fraction exhibited documented reactions with the parent compounds (Figure 3A).

### Characterization and Prioritization of Transformation Products:

To assess the biological plausibility of the predicted products, their ADME (Absorption, Distribution, Metabolism, and Excretion) and toxicity properties were calculated. This step is critical because transformation can result in metabolites that are significantly more or less toxic than the parent compound. For organophosphate insecticides, for instance, metabolic activation via in-vivo oxidation by cytochrome P450 enzymes often leads to the formation of oxon metabolites, which are the biologically active forms that inhibit the critical enzyme acetylcholinesterase. These oxon metabolites are extremely potent AChE inhibitors, leading to the accumulation of the neurotransmitter acetylcholine causing the characteristic neurotoxic effects associated with organophosphate poisoning. The presented workflow is capable of predicting these exact types of oxidative transformations, thereby identifying the specific products responsible for the compounds’ primary mechanism of toxicity. In contrast, other metabolic pathways, such as hydrolysis, lead to detoxification. The ability to predict products from both activating and deactivating pathways is essential for understanding the net toxicological outcome of an exposure. We further prioritized products that may have undesirable characteristics and biological plausibility (https://zenodo.org/records/15881389, Table 6). Over 1,200 unique products fell into the top 1st percentile for at least one adverse ADME/Tox property (Figure 3B and Figure S3), flagging them as candidates for future experimental validation. The radar chart in Figure 3D visually summarizes how in-silico transformation can alter key physicochemical properties between a parent compound and its predicted product, such as Bioavailability, Molecular Weight, and predicted Drug-Induced Liver Injury. A predicted increase in DILI potential is concerning (Figure 3C and Figure S4), as the liver is the primary site of xenobiotic metabolism, and the formation of reactive electrophilic metabolites is a well-established mechanism for chemical-induced hepatotoxicity. This detailed comparison can aid in toxicology and risk assessment, as the ultimate toxic effect of a pesticide is a function of the concentration of its bioactive form (the parent or a metabolite) at the target site. The generation of this expanded library of potential transformation products provides a key resource for improving biomonitoring and enhancing our understanding of insecticide exposure.

## Discussion:

This study presents a new workflow that utilizes >90,000 entries of PubChem reaction data to generate templates for in-silico transformation analysis. By applying this workflow to 181 insecticides, we predicted 21,284 unique transformation products, substantially expanding the known chemical space for this important class of environmental exposures. A key finding was that while 1,304 of these products were known compounds in PubChem, many had not been previously associated with their parent insecticides, representing new, high-confidence transformation hypotheses. Furthermore, the generation of thousands of entirely new chemical structures highlights the workflow’s potential to illuminate the “dark matter” of the chemical exposome. The integration of ADME-Tox property prediction provides a prioritization approach, allowing researchers to refine this large list of potential products into a smaller, manageable list of high-priority candidates for further investigation by biomonitoring and toxicity studies.

To demonstrate the utility of the workflow, a chemically diverse set of 181 insecticides was selected to investigate their potential transformations. This addresses a gap in existing literature which has largely focused only on parent compounds rather than their transformation products. Insecticides are used widely in agricultural, domestic, and public health settings. While they are crucial for crop yield, there is growing concern over their effects on non-target organisms and the environment^29–31^. Human exposure occurs through various routes, including occupational handling and low-level chronic exposure from food, water, and household dust. Human biomonitoring studies have confirmed ubiquitous exposure to classes like organophosphates and pyrethroids by measuring their metabolites in urine^29, 32^. The product database generated in this work can directly enhance these biomonitoring efforts by expanding targeted screening panels and improving the interpretation of untargeted datasets where metabolites often correlate with parent chemicals.

Our study has introduced the application of the reaction data from PubChem^19^ in creating a template library of reactions and applied to an important chemical class in the exposome. This extensive data set enabled the extraction of a diverse array of transformation templates, capturing a broad spectrum of known chemical and biological reactions. These templates facilitated the systematic prediction of metabolites by matching the reactive sites of insecticides with known reaction patterns, allowing for comprehensive simulation of potential transformation products. Each predicted metabolite was subsequently queried against the PubChem database to determine whether it corresponded to a known compound or represented a novel, previously uncharacterized entity.

By integrating transformation prediction with ADME-Tox profiling, this study provides a useful strategy to illuminate the “dark matter” of the chemical exposome, the vast number of uncharacterized transformation products that represent a significant gap in exposure science. The large list of 21,284 potential products for insecticides is computationally divided into a much smaller, manageable list of high-priority candidates ranked by biological plausibility and potential hazard. This tiered approach provides a data-driven guide for experimental toxicologists and analytical chemists, directing limited resources toward synthesizing and testing the specific products most likely to be a risk to human health.

Our study has few limitations. PubChem reaction data is crowd-sourced so there might be reactions with missing reactants or incorrect structures. We did observed that 1.56% of the predicted products are already cataloged in PubChem, supporting fidelity to the underlying reaction templates. However, the biological significance of the thousands of novel products remains to be determined. The critical next step is to leverage these predictions to query existing untargeted metabolomics data from human cohorts, animal models, or environmental samples. Finding evidence of these computationally predicted metabolites in real-world biospecimens would provide strong validation for the workflow and confirm the presence and significance of these new components of the chemical exposome.

## Conclusion:

We have integrated existing reaction data and cheminformatics software to develop a new workflow that can predict the transformation products for an exposome chemical. We have demonstrated the application of this in-silico transformation analysis (ISTA) workflow to expand the chemical space of insecticides. This workflow can expand the chemical exposome databases by many folds and thus will allow us to better understand the role of environmental toxins and chemicals on human health.

## Supplementary Material

Supplement 1

**Supporting information:** Additional tables and figures are available in the SI section

## Data Availability

Data are available at Zenodo https://zenodo.org/records/15881389
